# A Systematic Scoping Review of New Attention Problems Following Traumatic Brain Injury in Children

**DOI:** 10.3389/fneur.2021.751736

**Published:** 2021-11-10

**Authors:** Sonja Stojanovski, Shannon E. Scratch, Benjamin T. Dunkley, Russell Schachar, Anne L. Wheeler

**Affiliations:** ^1^SickKids Research Institute, Program in Neuroscience and Mental Health, Hospital for Sick Children, Neuroscience and Mental Health Program, Toronto, ON, Canada; ^2^Physiology Department, University of Toronto, Toronto, ON, Canada; ^3^Bloorview Research Institute, Holland Bloorview Kids Rehabilitation Hospital, Toronto, ON, Canada; ^4^Rehabilitation Sciences Institute, University of Toronto, Toronto, ON, Canada; ^5^Department of Paediatrics, University of Toronto, Toronto, ON, Canada; ^6^Institute of Medical Science, University of Toronto, Toronto, ON, Canada; ^7^Medical Imaging, University of Toronto, Toronto, ON, Canada; ^8^Psychiatry Department, University of Toronto, Toronto, ON, Canada

**Keywords:** ADHD, traumatic brain injury, attention, secondary ADHD, pediatric

## Abstract

**Objective:** To summarize existing knowledge about the characteristics of attention problems secondary to traumatic brain injuries (TBI) of all severities in children.

**Methods:** Computerized databases PubMed and PsychINFO and gray literature sources were used to identify relevant studies. Search terms were selected to identify original research examining new ADHD diagnosis or attention problems after TBI in children. Studies were included if they investigated any severity of TBI, assessed attention or ADHD after brain injury, investigated children as a primary or sub-analysis, and controlled for or excluded participants with preinjury ADHD or attention problems.

**Results:** Thirty-nine studies were included in the review. Studies examined the prevalence of and risk factors for new attention problems and ADHD following TBI in children as well as behavioral and neuropsychological factors associated with these attention problems. Studies report a wide range of prevalence rates of new ADHD diagnosis or attention problems after TBI. Evidence indicates that more severe injury, injury in early childhood, or preinjury adaptive functioning problems, increases the risk for new ADHD and attention problems after TBI and both sexes appear to be equally vulnerable. Further, literature suggests that cases of new ADHD often co-occurs with neuropsychiatric impairment in other domains. Identified gaps in our understanding of new attention problems and ADHD include if mild TBI, the most common type of injury, increases risk and what brain abnormalities are associated with the emergence of these problems.

**Conclusion:** This scoping review describes existing studies of new attention problems and ADHD following TBI in children and highlights important risk factors and comorbidities. Important future research directions are identified that will inform the extent of this outcome across TBI severities, its neural basis and points of intervention to minimize its impact.

## Introduction

Traumatic brain injury (TBI) is a major cause of acquired disability in children (1), and altogether accounts for over 700,000 emergency room visits annually in the United States (2). Importantly, emergency room visits underreport the true incidence of TBI as the majority of injuries are at the mild end of the spectrum (referred to as mild TBI or concussion) and children with mild injuries may seek medical attention from family doctors, other medical professions or not at all. The high prevalence of mild injuries is supported by surveys of high school students in which one in five adolescents report a lifetime history of concussion (3, 4).

TBI in children is associated with many different types of adverse outcomes as TBI to the developing brain impacts ongoing developmental processes (5). These include behavioral (6) and social problems (7), difficulties with academic achievement (8), and persistent cognitive deficits (9) that can occur across the spectrum of severities. The literature suggests a dose-response relationship between severity and outcome. Children with mild TBI typically recover within a month of injury (10) and the poorest outcomes, which may worsen over time, are seen in severe TBI (11, 12). Attention problems in particular are thought to be a common sequela of TBI (13). Attention supports higher cognitive thinking, learning, and problem-solving, and attention problems can negatively impact social interactions and the ability to function well at home and in school (14, 15). Attention problems that arise along with hyperactivity and impulsivity after TBI may contribute to new diagnoses of attention deficit hyperactivity disorder (ADHD) (16). The onset of ADHD symptoms (inattention, hyperactivity, and impulsivity) after an injury will hereafter referred to as secondary ADHD (SADHD) to be consistent with the majority of the existing literature. Importantly, the similarities and differences between primary and SADHD in youth are not fully understood and may represent very different disorders. This scoping review endeavors to begin to shed light on these differences by better characterizing SADHD which may inform the need for updated nomenclature. Studies have estimated that the rates of SADHD diagnoses may be as high as 46% in children hospitalized for TBI (17). Importantly, ADHD is also a risk factor for TBI (16), as children with ADHD are more likely to be injured, so disentangling preexisting ADHD from SADHD is important for understanding the etiology of attention problems and ADHD in children with TBI.

We identified six existing systematic reviews that included examinations of ADHD or attention problems after pediatric TBI (18–23). Generally, these reviews report that attention problems are among the most commonly reported problems in individuals with a history of brain injury. However, none of the existing reviews sought to explicitly characterize new attention problems or SADHD by accounting for preinjury attention problems or ADHD before the injury. Since the etiology of pre-existing ADHD and SADHD are likely to be different, assessing the existing literature that addresses SADHD specifically is necessary to guide future research that aims to determine its causes and provide treatment. A recent meta-analysis took the crucial step of controlling for or eliminating preinjury ADHD, but exclusively investigates whether there is an association between diagnosed ADHD and TBI (24). Given the dearth of synthesized literature capturing new attention problems, defined broadly, this review sought to formally assess what research evidence has been presented on the topic of attention problems that emerge after TBI in the literature to date. Systematic reviews and meta-analyses are important tools for addressing questions of prevalence or risk factors, however they have a narrow scope. Conversely, a scoping review framework was deemed most appropriate to achieve the goal of charting the data according to key general themes to identify knowledge gaps to guide focused research questions moving forward.

Here we review and synthesize the literature on attention problems acquired after TBI in children to summarize the existing knowledge base, as well as to identify areas requiring further research. We consider studies that examine attention problems specifically as well as those that assess SADHD diagnosis, though acknowledge that ADHD is a complex disorder that also includes problems with impulsivity and hyperactivity. Given the volume and scope of literature unearthed, extracted data was organized into categories which summarized: the prevalence of new attention problems and SADHD, injury and non-injury factors associated with risk for developing new attention problems as well as the behavioral presentation and associated neuropsychological impairments.

## Methods

The approach for this scoping review was guided by a protocol drafted using the Preferred Reporting Items for Systematic Reviews and Meta-analysis extension for scoping reviews (PRISMA-ScR) (25). The protocol which is available *via* the Open Science Framework (https://bit.ly/2YcIMYr) was submitted for registration in December 2020 to ensure transparency. To be included in the review, sources needed to formally assess symptoms of ADHD or measure attention problems following TBI in children (age 0–18 years).

We conducted a scoping review of scholarly and gray literature. To identify potentially relevant scholarly studies, PubMed and PsycINFO were searched from inception to June 2021. The final search strategy used the following search terms to search in title and abstract text in scholarly literature: “ADHD” or “attention deficit disorder” or “attention deficit hyperactivity” and “secondary” or “after” or “following” and “concussion” or “concussions” or “concussive” or “TBI” or “TBIs” or “mTBI” or “mTBIs” or “brain injury” or “brain injuries” or “brain damage” or “head impacts” (see Appendix S1 for the search strategy applied to PubMed). The study design filter was set to select only peer-reviewed journal articles in the PsycINFO search. The literature search was performed by SS, in consultation with AW. The final search results were exported into Zotero, before being imported into Covidence where duplicates were removed.

Additionally, gray literature, *via* vital statistics data, government surveillance data and reports, Centers for Disease Control and Prevention data, population censuses and surveys (i.e., national or provincial health survey data) and disease association websites were sought to decrease publication bias, and introduce alternative perspectives (26). Following the identification of relevant authorities these sources were searched through targeted website searching. Where targeted website searching turned up no sources advanced searching was applied to search the websites of the relevant authorities. The gray literature search applied the same search terms utilized for the scholarly literature search.

The literature search was performed by SSt and AW. To ensure consistency of screening 10 titles and abstracts were selected at random, independently screened and results were discussed to amend the screening protocol. All titles and abstracts were subsequently evaluated. Literature was retained for full-text search if it was: written in English, involved human participants, presented primary research, investigated any severity of TBI, assessed attention or ADHD after brain injury, and investigated children as a primary or sub-analysis. We required that studies controlled for or excluded participants with preinjury ADHD or attention problems to isolate attention outcomes secondary to TBI. Full texts of publications identified by the search and screening were independently assessed and retained for extraction if they contained the same features as were considered for the title and abstract screening. Disagreements on study selection were resolved by discussion if needed.

A data charting form was jointly developed by SSt and AW to determine which variables to extract and piloted on five randomly selected papers that passed full text screening. The two reviewers then iteratively updated the data-charting form to be applied to all eligible studies. Data charting was performed by SSt and verified by AW for accuracy, and inconsistencies were resolved through discussion. Specifically, data was abstracted on the following study characteristics: study design, study population, sample sizes, control groups, brain injury characteristics, and contextual factors (i.e., injury severity, age at injury, time since injury), how preinjury attention was controlled for, and how attention was assessed. Additionally, we abstracted data related to the following domains: prevalence, risk factors, behavioral features (including comorbidities), and neuropsychological characteristics associated with secondary attention problems and ADHD. Attention problems were defined according to thresholds for clinical significance in the individual scales used in each publication. We then grouped studies by the domain(s) of interest that they assessed and summarized the results of those analyses within each study.

## Results

**Figure 1:**
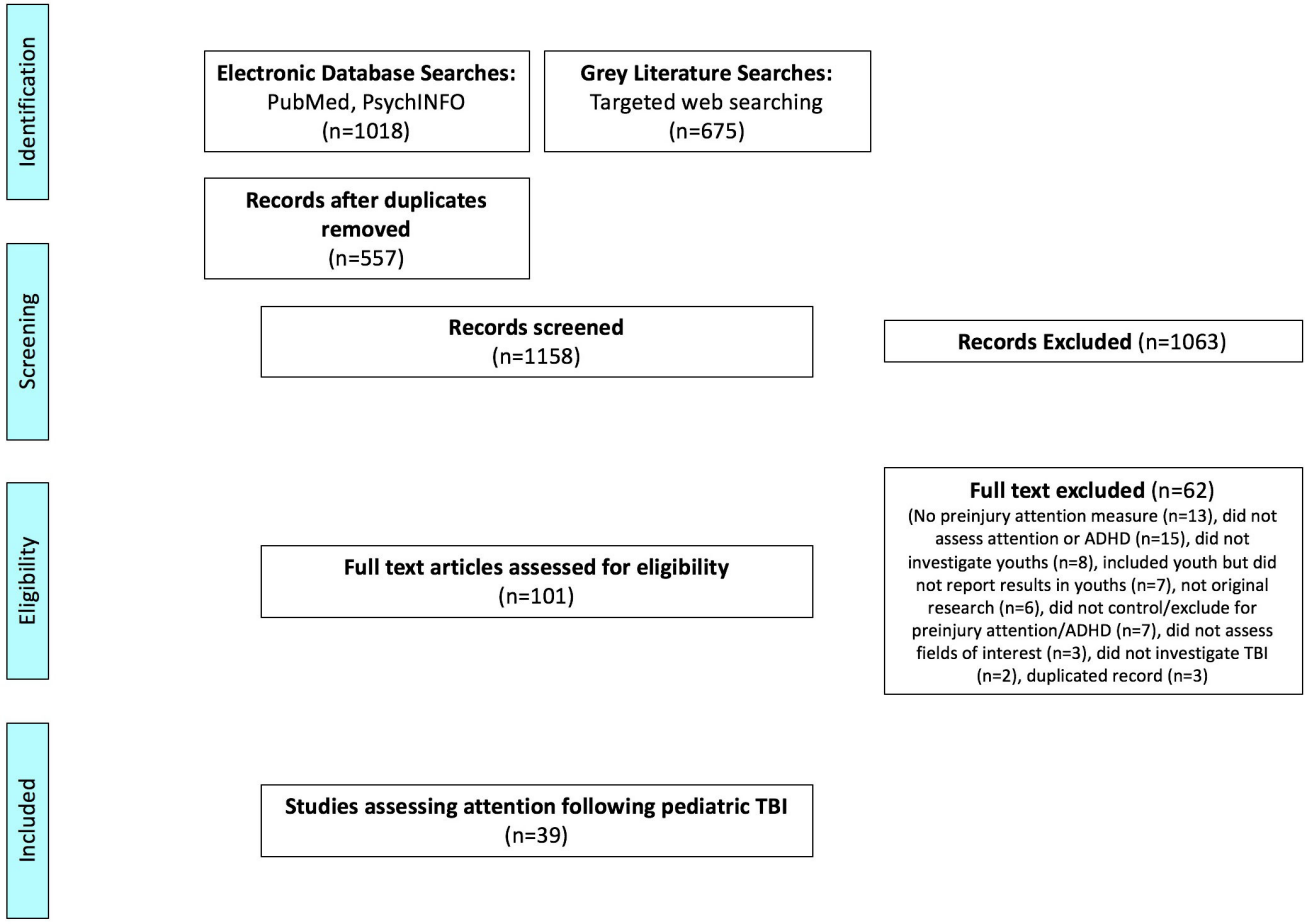
Flowchart article selection procedure.

After duplicates were removed, a total of 557 citations were identified from searches of electronic databases. Additionally, 675 citations were identified from searches of gray literature. Based on title and abstract, 1,158 were excluded and 101 full-text articles were retrieved and assessed for eligibility. Of these 13 were excluded for having no estimate of preinjury attention, 15 did not assess attention or ADHD, eight did not investigate children, seven studies included children but did not report results in children, six were not original research articles, seven did not control or exclude for preinjury attention or ADHD, three were excluded for assessing no fields of interest (prevalence, risk factor, behavioral features, or neuropsychological impairment), two did not investigate TBI, and three were excluded for being a duplicate. The remaining 39 studies were included in this review (Figure 1).

### Study Characteristics

An overview of the characteristics of each of the 39 studies is provided in Table 1. All 39 studies were derived from scholarly literature while no material from the gray literature search survived abstract and full text screening. Of the 39 studies more than half, 24, were prospective cohort studies of which two were birth cohorts. Twelve of the studies were retrospective studies, eight were cross-sectional, two were population cohorts, and two were chart reviews. Two studies combined prospective and retrospective cohorts. The majority of the studies (25) examined youth across the spectrum of TBI severity: mild, moderate, and severe TBI. Of these studies, five restricted the mild cases in their sample to those that were classified as “complicated mild,” meaning that there were positive findings on clinical neuroimaging assessments. Six studies included only moderate and severe cases of TBI, two included only severe TBI, and six included only mild TBI, including one that was a sample of children with a concussion. Nineteen studies included only youth with TBI whereas 20 studies also had a non-TBI comparison group, 10 were orthopedic injury controls and 10 had comparison groups that did not have a TBI. The range in age of injury of the subjects in each sample varied with more representation of older children and adolescents than younger children; only six studies included children injured in the first 3 years of life whereas 10 studies included older adolescents, age 16–18. The longest post-injury assessment time points in the studies ranged from 3 months to 20 years with six studies assessing youth less than a year after their injury, nine between 1 and 2 years, 11 between 2 and 5 years, nine between 5 and 10 years, and four longer than 10 years post-injury. The majority of study samples were hospitalized youth (27), whereas one study recruited only youth who visited the emergency department, three were recruited from TBI clinics and five studies included youth recruited from a combination of these settings. Only three studies examined population-based samples. Approximately half of the studies (20) assessed ADHD diagnosis as the attention outcome, whereas eight studies used an attention problem scale, and five studies used an attention task. Six studies used a combination of diagnosis, symptoms, scales, and tasks to assess attention. As this review includes only studies that assessed ADHD that is secondary to a TBI, studies used different approaches to account for preinjury attention. The majority of the studies (22) excluded youth with pre-injury (i.e., primary) diagnoses of ADHD. Eleven studies controlled for preinjury attention by including a retrospective assessment as a covariate in their statistical models, four studies examined youth with primary ADHD separately and two studies were birth cohorts which allowed them to establish the emergence of attention problems after injury in early childhood.

**Table 1:** Characteristics of included studies.

**References**	**Study design**	**TBI severity**	**Groups and Ns**	**Age at injury**	**Injury to assessment interval(s)**	**Population**	**Attention assessment(s)**	**How was preinjury ADHD controlled for?**
Yeates et al. (28)	Prospective cohort	Moderate, severe	82 TBI; 50 OI	6–12 yrs	4 yrs	Hospitalized	CBCL attention scale; ADHD scale, Focused Attention Task	Baseline attention problems as a covariate
Yang et al. (29)	Retrospective (population) cohort	Mild, moderate, severe	10,416 TBI; 41,664 no TBI	0–12 yrs	9 yrs	Population	ADHD Diagnosis	P-ADHD excluded
Wilkinson et al. (30)	Prospective cohort	Mild, moderate, severe	58 TBI	5–17 yrs	3 mo; 6 mo; 1 yr	Hospitalized	Conners-3 ADHD scales	Baseline attention problems as a covariate
Wassenberg et al. (31)	Prospective cohort	Mild, moderate, severe	42 TBI	6–14 yrs	2w; 3 mo; 6 mo; 1 yr; 2 yrs	Hospitalized	ADHD Diagnosis; Sustained Attention Task	P-ADHD excluded
Wade et al. (32)	Prospective cohort	Complicated mild, moderate, severe	80 TBI; 113 OI	3–7 yrs	6 mo	Hospitalized	ADHD Diagnosis; CBCL ADHD scale	Baseline attention problems as a covariate
Vasa et al. (33)	Prospective cohort	Severe	97 TBI	4–19 yrs	1 yr	Hospitalized	ADHD Diagnosis	P-ADHD excluded
Treble-Barna (34)	Prospective cohort	Complicated mild, moderate, severe	72 TBI; 95 OI	3–7 yrs	6 mo; 12 mo; 18 mo	Hospitalized	ADHD Diagnosis; CBCL ADHD scale	Baseline attention problems as a covariate
Studer et al. (35)	Prospective cohort	Mild	40 TBI; 38 OI	6–16 yrs	1 w 1 mo 4 mo	ED	Studer every day attention scale	Baseline attention problems as a covariate
Slomine et al. (36)	Retrospective cross sectional	Severe	22 TBI	6–16 yrs	1 yr	Hospitalized	ADHD Diagnosis, Attention Task	P-ADHD examined separately
Sinopoli et al. (37)	Retrospective cross sectional	Mild, moderate, severe	44 no TBI no ADHD; 19 no TBI ADHD; 40 TBI no ADHD; 9 TBI ADHD	7–17 yrs	1–6 yrs	Hospitalized	ADHD Diagnosis	P-ADHD excluded
Schachar et al. (38)	Retrospective cross sectional	Mild, moderate, severe	137 TBI; 63 no TBI	5–17 yrs	2–15 yrs	Hospitalized	ADHD Diagnosis	P-ADHD excluded
Power et al. (39)	Prospective cohort	Moderate, severe	36 TBI	6–14 yrs	5 yrs	Hospitalized	Attention Capacity and Attention Control Tasks	P-ADHD excluded
Ornstein et al. (40)	Prospective cohort	Mild, moderate, severe	26 TBI P-ADHD; 17 TBI S-ADHD; 98 TBI no ADHD	6–16 yrs	6 mo; 12 mo	Hospitalized	ADHD Diagnosis, Divided attention task	P-ADHD examined separately
Ornstein et al. (41)	Retrospective cross sectional	Mild, moderate, severe	92 no TBI ADHD; 103 TBI; 79 no TBI no ADHD	6–14 yrs	Mean = 4.9 yrs, SD = 1.8 yrs^*^	ED; hospitalized	ADHD Diagnosis	P-ADHD excluded
Narad et al. (17)	Prospective cohort	Complicated mild, moderate, severe	54 TBI; 66 OI	3–7 yrs	6 mo; 12 mo; 18 mo; 3.4 yrs; 6.8 yrs	Hospitalized	ADHD Diagnosis	P-ADHD excluded
Narad et al. (42)	prospective cohort	complicated mild, moderate, severe	81 TBI; 106 OI	3-7 yrs	6 mo; 12 mo; 18 mo; 3.4 yrs; 6.8 yrs	hospitalized	ADHD Diagnosis	P-ADHD excluded
McKinlay et al. (43)	Prospective birth cohort	Mild	19 hospitalized TBI; 57 GP/ED TBI; 839 no TBI	0–5 yrs	11–16 yrs	ED; GP; hospitalized	ADHD Diagnosis	birth cohort
McKinlay (44)	Prospective birth cohort	Mild	26 hospitalized TBI; 65 GP/ED TBI; 839 no TBI	0–5 yrs	2–7 yrs, then 1/yr for 7 yrs	ED; GP; hospitalized	Conners-3 ADHD scale	birth cohort
Max et al. (45)	Retrospective chart review	Mild, moderate, severe	54 TBI	Mean = 6.1 yrs, SD = 4.9 yrs^*^	Mean = 5.3 yrs, SD = 4.6 yrs^*^	TBI clinic	ADHD Diagnosis	P-ADHD excluded
Max et al. (46)	Prospective cohort	Mild, moderate, severe	50 TBI	6–14 yrs	3 mo; 6 mo; 1 yr; 2 yrs	Hospitalized	ADHD Diagnosis	P-ADHD excluded
Max et al. (47)	Prospective and retrospective cohorts	Mild, moderate, severe	94 TBI	5–14 yrs	3 mo; 6 mo; 1 yr; 2 yrs	Hospitalized	ADHD Diagnosis	P-ADHD excluded
Max et al. (48)	Prospective and retrospective cohorts	Mild, moderate, severe	94 TBI; 24 OI	5–14 yrs	3 mo; 6 mo; 1 yr; 2 yrs	Hospitalized	ADHD Diagnosis	P-ADHD excluded
Max et al. (49)	Prospective cohort	Mild, moderate, severe	143 TBI	5–14 yrs	6 mo	Hospitalized	ADHD Diagnosis	P-ADHD excluded
Max et al. (27)	Prospective cohort	Mild, moderate, severe	103 TBI	5–14 yrs	6 mo-1 yr; 1–2 yrs	Hospitalized	ADHD Diagnosis	P-ADHD excluded
Max et al. (50)	prospective cohort	mild, moderate, severe	141 TBI	5-14 yrs	6 mo	hospitalized	ADHD Diagnosis	P-ADHD excluded
Li et al. (51)	Prospective cohort	Mild	57 single TBI; 42 multiple TBI; 319 no TBI	6 yrs	6 yrs	Population	CBCL ADHD scale	Baseline attention problems as a covariate
Levin et al. (52)	Prospective cohort	Mild, moderate, severe	114 TBI no P-ADHD; 34 TBI P-ADHD	5–15 yrs	6 mo; 1 yr; 2 yrs	Hospitalized	ADHD Diagnosis; K-SADS-PL ADHD scale	P-ADHD examined separately
Konrad et al. (53)	Retrospective cross sectional	Moderate, severe	13 TBI S-ADHD; 14 TBI no ADHD	8–12 yrs	6 mo - 6 yrs	TBI clinic	ADHD Diagnosis	P-ADHD excluded
Keenan et al. (54)	Prospective cohort	Mild, moderate, severe	386 TBI; 133 OI	2.5–15 yrs	3 mo; 12 mo	ED; hospitalized	CBCL ADHD scale	Baseline attention problems as a covariate
Karver (55)	Prospective cohort	Complicated mild, moderate, severe	68 TBI; 75 OI	3–7 yrs	Mean = 38.3 mo; SD = 10.3 mo^*^	Hospitalized	CBCL ADHD scale	Baseline attention problems as a covariate
Gerring et al. (56)	Prospective cohort	Moderate, severe	65 TBI no ADHD; 15 TBI S-ADHD	4–19 yrs	1 yr	TBI clinic; hospitalized	ADHD Diagnosis	P-ADHD excluded
Ellis et al. (57)	Retrospective chart review	Concussion	20 TBI PCS; 154 TBI no PCS	Mean 14.2 yrs, SD = 2.3 yrs^*^	Varied, at 1–4 weeks intervals	Sports concussion clinic	ADHD Diagnosis	P-ADHD examined separately
Chapman et al. (58)	Prospective cohort	Moderate, severe	76 TBI; 90 OI	3–7 yrs	6 mo; 12 mo; 18 mo	Hospitalized	CBCL ADHD scale	Baseline attention problems as a covariate
Chang et al. (59)	Retrospective (population) cohort	Mild, moderate, severe	8,801 TBI; 31,294 no TBI	0–3 yrs	10–20 yrs	Population	ADHD Diagnosis	P-ADHD excluded
Catroppa and Anderson (60)	Retrospective cross sectional	Mild, moderate, severe	76 TBI	8–12 yrs	3 mo	Hospitalized	Selective and sustained attention task (CPT)	P-ADHD excluded
Catale et al. (61)	Retrospective cross sectional	Mild	15 TBI; 15 no TBI	6–12 yrs	1 yr	Hospitalized	Attention task battery	Baseline attention problems as a covariate
Bloom (62)	Retrospective cross sectional	Mild, moderate, severe	46 TBI	6–15 yrs	>1 yr (2–3 on average)	Hospitalized	ADHD Diagnosis	P-ADHD excluded
Anderson et al. (63)	Prospective cohort	Mild, moderate, severe	56 TBI; 26 no TBI	2–7 yrs	30 mo	Hospitalized	Selective and sustained attention task (CPT)	Baseline attention problems as a covariate

*OI, orthopedic injury; OFC, orbitofrontal cortex; SADHD, secondary ADHD; PADHD, primary ADHD; mo, months; yr(s), year(s); GP, General Practitioner; ED, emergency department; CPT, continuous performance task; CBCL, child behavior checklist*.

* *For Age at Injury and Injury to Assessment Interval(s) range was reported when they were available, but mean and SD were reported when they were not*.

### Prevalence of ADHD and Attention Problems Following TBI

We consider studies that examine attention problems specifically as well as those that assess SADHD. Eighteen studies reported the prevalence of SADHD after TBI and these are reported at the longest measured timepoint for each study in Table 2. Two studies reported the prevalence of new attention problems after TBI and these are reported hereafter.

**Table 2:** Studies of prevalence.

**References**	**TBI severity**	**Age at injury**	**Longest assessment timepoint**	**SADHD at longest assessment timepoint**
Ellis et al. (57)	Concussion	Mean 14.2 yrs, SD = 2.3 yrs^*^	1 yr	0%
McKinlay et al. (43)	Mild	0–5 yrs	9–16 yrs	21% hospitalized TBI; 11% GP/ED TBI; 6% noTBI
Yang et al. (29)	Mild, moderate, severe	0–12 yrs	9 yrs	5% TBI; 4% no TBI
Sinopoli et al. (37)	Mild, moderate, severe	7–17 yrs	6 yrs	18% TBI
Schachar et al. (38)	Mild, moderate, severe	5–17 yrs	15 yrs	36% TBI; 12% no TBI
Max et al. (45)	Mild, moderate, severe	Mean = 6.1 yrs, SD = 4.9 yrs^*^	2 yrs	53% TBI
Max et al. (48)^#^	Mild, moderate, severe	5–14 yrs	2 yrs	38% severe TBI; 12% moderate TBI; 10% mild TBI; 5% OI
Max et al. (49)^#^	Mild, moderate, severe	5–14 yrs	6 mo	16% TBI
Max et al. (27)^#^	Mild, moderate, severe	5–14 yrs	2 yrs	21% TBI
Chang et al. (59)	Mild, moderate, severe	0–3 yrs	> 10 yrs	6% TBI; 4% no TBI
Bloom (62)	Mild, moderate, severe	6–15 yrs	1 yr	44% TBI
Ornstein et al. (40)	Mild, moderate, severe	6–16 yrs	1 yr	11% TBI
Wassenberg et al. (31)	Mild, moderate, severe	6–14 yrs	2 yrs	17% TBI
Levin et al. (52)	Mild, moderate, severe	5–15 yrs	2 yrs	18% TBI
Narad et al. (17)^$^	Complicated mild, moderate, severe	3–7 yrs	6.8 yrs	46% TBI; 14% OI
Narad et al. (42)^$^	Complicated mild, moderate, severe	3–7 yrs	6.8 yrs	62% severe TBI; 30% moderate TBI; 33% complicated mild TBI; 15% OI
Vasa et al. (33)	Severe	4–19 yrs	1 yr	9% TBI
Slomine et al. (36)	Severe	6–16 yrs	1 yr	17% TBI

*SADHD, secondary ADHD; OI, orthopedic injury; mo, months; yr(s), year(s)*.

*SADHD at longest assessment timepoint: Presents prevalence of population, or subpopulations as a percentage*.

* *For Age at Injury range was reported when it was available, but mean and SD were reported when it was not*.

# *These samples overlap with one another*.

$ *These samples overlap with one another*.

Two of these studies reported prevalence at different time points post-injury from an overlapping sample (27, 49) and two studies reported a breakdown of prevalence by injury severity (42, 48) in samples that overlapped with samples from studies where they reported the prevalence for all severities (17, 27, 49). Nine studies included a control comparison group that allowed for statistical comparisons. In studies that examined the full range of TBI severity the prevalence of ADHD diagnosis after TBI ranged from 5 to 53%. Prevalence was lowest in population-based studies (29, 59), though notably, these rates were statistically higher than controls in those studies. Prevalence was highest in the one study that recruited from a TBI clinic, 53% (45). Prevalence ranged from 11 to 46% in hospitalized samples. Four studies examined either mild TBI only or reported prevalence in mild TBI specifically. A study in a concussion sample reported no new ADHD post injury (57), whereas, a prospective birth cohort of children who had sustained a mild injury in early childhood, reported that 11% of children who had been outpatients and 21% of children who had been inpatients at the time of injury were diagnosed with ADHD by mid-adolescence compared to 6% of children with no TBI (43). A mixed retrospective and prospective study reported that 10% of subjects with mild TBI developed ADHD compared to 5% of subjects with orthopedic injury (48) whereas a study in younger children reported 33% of subjects with complicated mild TBI developed ADHD compared to 15% of subjects with orthopedic injury (42). The same two studies reported prevalence of moderate TBI as 12% and severe TBI as 38% compared to 5% in orthopedic injury (48) in the first study, and 30% in moderate TBI and 62% in severe TBI compared to 15% in orthopedic injury (42) in the second study. Two studies examined severe TBI exclusively reporting 9% (33) and 17% (36) prevalence. Where statistical comparisons were possible the prevalence of SADHD after severe TBI was always significantly higher than the controls (28, 42, 48, 58), whereas this was not always the case for mild and moderate TBI (42, 48). Studies by Chapman et al. (58) and Yeates et al. (28) examined youth with moderate and severe TBI and reported clinically significant attention problems after severe injury (32 and 46%, respectively) compared to orthopedic injury controls (7 and 26%, respectively).

### Risk Factors

Twenty-five studies examined risk factors associated with children developing ADHD or new attention problems after TBI. We separated risk factors into injury-related and non-injury related and have summarized the significant and non-significant factors in Table 3.

**Table 3:** Studies of risk factors.

**References**	**TBI severity**	**Age at injury**	**Injury related risk factors (significant)**	**Injury related risk factor (not significant)**	**Non-injury related risk factor (significant)**	**Non-injury related risk factor (not significant)**
Li et al. (51)	Mild	6 yrs	More than 1 injury	Single injury		
McKinlay (44)	Mild	0–5 yrs	Mild inpatient	Mild outpatient		
Ornstein et al. (41)	Mild, moderate, severe	6–14 yrs		Time since injury, GCS		Age at injury, age at time of assessment, sex
Schachar et al. (38)	Mild, moderate, severe	5–17 yrs	Higher severity	Time since injury	Greater preinjury behavior problems	Age at injury, age at time of assessment, sex
Max et al. (46)	Mild, moderate, severe	6–14 yrs	Earlier time post injury (mild TBI), CT scan bicaudate ratio at 6 & 12 months post injury	Time post injury, GCS, CT scan bicaudate ratio and bifrontal ratio at 3 & 24 months post injury	Poorer family function	SES, family psychiatric history, age at injury, litigation status, sex
Max et al. (48)	Mild, moderate, severe	5–14 yrs	Higher severity, lower GCS, time LOC	CT lesion area	Poorer family function	Age at injury, age at assessment, sex, preinjury psychiatric disorder, family psychiatric history, family strain, family history of ADHD, SES
Max et al. (49)	Mild, moderate, severe	5–14 yrs	Lesion on T1 in OFC at 6mo	GCS, lesion on T1 in non-OFC regions at 6mo	Lower SES, lower preinjury adaptive function	Age at injury, sex, race, preinjury lifetime psychiatric disorder, preinjury family function, family psychiatric history, family history of ADHD in first-degree relatives,
Max et al. (27)	Mild, moderate, severe	5–14 yrs	Lesion on T1 in cerebellum at 6-12 mo	GCS, lesion on T1 outside cerebellum (6–12 mo), lesions on T1 in all regions (12–24 mo)	Lower SES, lower preinjury adaptive function, higher psychosocial adversity	Age at injury, sex, race, preinjury lifetime psychiatric disorder, preinjury family function, family psychiatric history, family history of ADHD in first-degree relatives
Yang et al. (29)	Mild, moderate, severe	0–12 yrs	Contusion, subdural hemorrhage	Skull fracture; subarachnoid hemorrhage; epidural hemorrhage; intracerebral hemorrhage	Younger age of injury (<8)	
Chang et al. (59)	Mild, moderate, severe	0–3 yrs	Higher severity of injury, more than 1 injury		Low birth weight, age (<1)	Perinatal infection, maternal perinatal morbidity, birth trauma, hypoxia/birth asphyxia
Max et al. (45)	Mild, moderate, severe	Mean = 6.1 yrs, SD = 4.9 yrs^*^		Severity of injury		Family function, family history of alcohol dependence/abuse, Performance IQ, language rating, learning disability rating, age at injury
Ornstein et al. (40)	Mild, moderate, severe	6–16 yrs		GCS	Lower SES (6 mo)	Sex, age at injury, age of assessment, verbal IQ, SES (1 yr)
Levin et al. (52)	Mild, moderate, severe	5–15 yrs		Severity of injury	Lower SES	Age at injury, sex
Wassenberg et al. (31)	Mild, moderate, severe	6–14 yrs	Attention Task: shorter time since injury		Attention Task: lower pre-injury adaptive function, worse social background ADHD Diagnosis: early (2w) omission/inattention	ADHD Diagnosis: early (2w) commission/impulsiveness
Keenan et al. (54)	Mild, moderate, severe	2.5–15 yrs	Higher severity		Younger age (preschool), female sex	Family function, social capital
Wilkinson et al. (30)	Mild, moderate, severe	5–17 yrs	Longer time since injury	GCS		Age at injury, sex
Anderson et al. (63)	mild, moderate, severe	2–7 yrs		severity of injury, lesion site	younger age at injury, preinjury behavior problems	preinjury adaptive function
Narad et al. (42)	complicated mild, moderate, severe	3–7 yrs			lower maternal education, greater poorer family function	age at injury, sex
Wade, 2011	complicated mild, moderate, severe	3–7 yrs			severe TBI - less post injury parental warm responsiveness, more baseline parental negativity	caregiver distress, family function, IQ, baseline parental warm responsiveness, moderate TBI - post injury parental warm responsiveness, post injury parental negativity, baseline parental negativity
Treble-Barna, (34)	complicated mild, moderate, severe	3–7 yrs	higher severity		severe TBI - baseline parental warm responsiveness	severe TBI - 12 mo parental warm responsiveness, age at injury, scaffolds, restrictive behaviors
Gerring et al. (56)	Moderate, severe	4–19 yrs	MRI lesions of the thalamus and/or basal ganglia 3 mo after injury	GCS, MRI lesions in the frontal cortex 3 mo after injury		
Chapman et al. (58)	Moderate, severe	3–7 yrs	Higher severity		Severe TBI—more preinjury attention problems	
Yeates et al. (28)	Moderate, severe	6–12 yrs			Severe TBI—more preinjury attention problems	moderate TBI—preinjury attention
Ekinci (64)	Moderate, severe	6–18 yrs		Severity of injury		
Power et al. (39)	Moderate, severe	6–14 yrs		Presence and location/severity of cerebellar lesion T1/T2 at 5 yrs post injury		
Slomine et al. (36)	Severe	6–16 yrs		GCS		SES, sex, ethnicity, age at injury

*OFC, orbitofrontal cortex; mo, months; yr(s), year(s); SES, socioeconomic status; GCS, Glasgow coma scale; LOC, loss of consciousness*.

* *For Age at Injury range was reported when it was available, but mean and SD were reported when it was not*.

### Injury Factors Associated With ADHD and Attention Problems Following TBI

As suggested by the prevalence rates grouped by severity, studies that examined severity as a moderating factor sometimes reported that increased severity based on grouping (mild, moderate, and severe) or Glasgow Coma Score (GCS), an index of severity based on the level of consciousness after an injury, was associated with higher rates of ADHD diagnosis or attention problems. However, an equal number of studies found that severity/GCS was not associated with SADHD or attention problems. Two studies examined the impact of having more than one injury and reported that this was associated with increased attention problems or SADHD diagnosis (51, 59). Only one study examined the loss of consciousness, reporting that increased time spent unconscious after the injury was associated with developing ADHD (48). Two prospective cohort studies with similar design reported an association between time since injury and attention problems, however with opposite directions of effect (30, 31). Two retrospective studies that assessed children many years after injury reported no effect of time since injury suggesting that attention problems do not get worse or improve over time after injury (38, 41).

The ability of neuroimaging to predict risk for subsequent new attention problems was investigated in several studies. Acute imaging with computerized tomography (CT) is common after injury and in their population study. Yang et al. (29) found that contusion and subdural hemorrhage, but not other types of hemorrhage or skull fracture were associated with subsequent ADHD diagnosis. A study by Max et al. (45) measured 2 frontal ratios and reported that bicaudate ratio, indicative of ventricular compression, was associated with attention problems when measured 6 and 12 months after injury but not at 3 months and 2 years post-injury. Another prospective cohort study by Max and colleagues employed magnetic resonance imaging (MRI) and demonstrated that lesions in the orbital frontal cortex 6 months after injury and the cerebellum 6–12 months after injury were associated with SADHD. However, these findings did not hold for the other time points examined and there were no relationships between lesions in other regions and SADHD (27). Another MRI study by Gerring et al. (56) found an association between lesions in the thalamus and basal ganglia 3 months after injury and SADHD, but no association with lesions in the frontal cortex. Power et al. (39) examined MRIs 5 years after injury and noted no relationship between cerebellar lesions or the location and severity of cerebral lesions and performance on attention tasks. Similarly, Anderson et al. (63) noted that there were no significant relationships between lesion site and attentional impairment after injury in a preschool-aged sample.

### Non-injury Factors Associated With ADHD and Attention Problems Following TBI

Most studies reported the influence of the age at the time of injury on the subsequent diagnosis of ADHD or new attention problems. For the most part, these studies did not report significant effects, however, the four studies that did report that younger children were at increased risk of attention problems were those that included the youngest children in their samples (29, 54, 59, 63). Of the 12 studies that examined sex as a possible risk factor all but one reported that there was no increased risk for attention problems for males or females (27, 30, 36, 38, 40–42, 46, 48, 49, 52). The study that did report an effect found that female sex increased the risk for attention problems (54). An equal number of studies found that socioeconomic status was a significant (27, 49, 52) or non-significant (36, 40, 45, 48) risk factor for new ADHD or attention problems. Worse family functioning was sometime (42, 45, 48) but not always (27, 32, 46, 49, 54) shown to be associated with ADHD or more attention problems. Wade et al. (32), assessed aspects of family function and reported that in severe but not moderate TBI more baseline parental negativity and less parental warmth and responsiveness were associated with more attention problems. The population study by Chang et al. (59) found that low birth weight but no other perinatal or birth factors increased the risk of developing ADHD after TBI. Studies that examined family psychiatric history (27, 45, 48, 49) or baseline IQ (32, 40, 46) did not find associations with subsequent attention problems following TBI. Lower preinjury adaptive functioning was reported as a significant factor in 3 out of 4 studies that assessed it (27, 31, 49, 63). Greater preinjury behavior problems (38, 63), as well as preinjury attention problems (28, 58) specifically in severe TBI, were consistently associated with secondary attention problems.

### Behavioral Features Associated With ADHD and Attention Problems Following TBI

Thirteen studies reported behavioral features of SADHD, though five of these studies examined overlapping samples. These behavioral features are summarized in Table 4. All studies were prospective cohort studies of hospitalized samples. Two studies described inattention as the predominant feature of SADHD. One study noted that the inattention but not hyperactivity/impulsivity subscales of the Conners-3 ADHD scale increased pre to post injury (30) and another reported the inattentive ADHD subtype was the most common while hyperactive symptoms declined over time in children with SADHD (52). Children with SADHD were reported to have reduced communication skills and socialization skills compared to children that did not develop SADHD as well as children with primary ADHD. However, ratings of daily living skills were not significantly different between groups (40). In line with this study, another study also found that SADHD was associated with reduced adaptive functioning as well as intellectual function relative to children who did not develop ADHD after injury (48). Another study found that SADHD was associated with worse functional impairment and behavioral regulation but not metacognition in comparison with children with TBI that do not develop ADHD, as well as children with an orthopedic injury that develop SADHD (17). The emergence of SADHD has also been associated with co-occurring novel psychiatric problems including oppositional defiant disorder and conduct disorder (27, 45, 49), and affective lability (27, 33, 47–50). In the same sample, SADHD did not co-occur with new-onset anxiety disorders but did co-occur with new-onset depressive disorders at a 1 yr but not 6 mo or 2 years post injury (27, 49).

**Table 4:** Studies of associated behavioral features.

**References**	**TBI severity**	**Age at injury**	**Behavioral presentation**
Wilkinson et al. (30)	Mild, moderate, severe	5–17 yrs	Inattention increased from baseline but not: hyperactivity, impulsivity, executive functioning, learning problems, defiance/aggression, and peer relations
Ornstein et al. (40)	Mild, moderate, severe	6–16 yrs	SADHD had reduced adaptive functioning, communication skills and socialization skills compared to no SADHD and PADHD
Max et al. (46)	Mild, moderate, severe	6–14 yrs	ADHD symptoms correlated with ODD symptoms at each assessment timepoint (baseline, 3, 6, 12, 24 months)
Max et al. (47)	Mild, moderate, severe	5–14 yrs	SADHD associated with personality change
Max et al. (48)	Mild, moderate, severe	5–14 yrs	SADHD associated with personality change
Max et al. (49)	Mild, moderate, severe	5–14 yrs	SADHD associated with new onset/secondary personality change and new ODD/CD but not new depressive disorder or new anxiety disorder at 6 months post injury
Max et al. (27)	Mild, moderate, severe	5–14 yrs	SADHD associated with new onset/secondary personality change and new ODD/CD but not new depressive disorder or new anxiety disorder at 12–24 months post injury
Max et al. (50)	Mild, moderate, severe	5–14 yrs	Comorbidity of SADHD and personality change
Levin et al. (52)	Mild, moderate, severe	5–15 yrs	The inattentive ADHD subtype was the most common subtype at follow-up in children without preinjury ADHD. Hyperactive symptoms declined over time in patients with SADHD
Max et al. (48)	mild, moderate, severe	5–14 yrs	SADHD associated with significant impairment in intellectual and adaptive function compared to no SADHD
Narad et al. (17)	Complicated mild, moderate, severe	3–7 yrs	SADHD associated with worse functional impairment, and behavioral regulation (behavioral control, emotional instability, and disinhibition) compared to no SADHD and OI with SADHD
Yeates et al. (28)	Moderate, severe	6–12 yrs	Cognitive tasks of attention related to CBCL attention and ADHD Rating Scale in TBI and OI
Vase (2015)	Severe	4–19 yrs	94% co-occurrence between SADHD and clinical affective lability

*yr(s), year(s); SADHD, secondary ADHD; PADHD, primary ADHD; OI, orthopedic injury; ODD, oppositional defiant disorder; CD, conduct disorder*.

### Neuropsychological Impairments

Unsurprisingly tests of attention revealed impaired function after TBI. One study found impaired selective attention after mild TBI (61), another found impaired focused attention after severe but not moderate TBI compared to controls (28) and two studies found that children with severe TBI perform more poorly than mild to moderate groups on a sustained attention task (60, 63). Children with SADHD performed worse on a divided attention task compared to children who did not develop ADHD after an injury as well as those that had primary ADHD (40). Though children with TBI were found to have deficits in inhibitory control processing in the stop-task similar to children with primary ADHD (53), there is mixed evidence as to if children with SADHD are especially impaired (37, 38, 40, 41, 53). New post-injury attention problems in children were associated with poorer verbal learning (35), short term memory (36), and working memory as well as planning ability (40). These neuropsychological impairments are summarized in Table 5.

**Table 5:** Studies of associated neuropsychological impairments.

**References**	**TBI severity**	**Age at injury**	**Neuropsychological impairment**
**ATTENTION TASKS**			
Catale et al. (61)	Mild	6–12 yrs	Mild TBI showed impaired selective attention
Catroppa and Anderson (60)	Mild, moderate, severe	8–12 yrs	Severe TBI perform more poorly than mild/moderate groups on a sustained attention task (CPT)
Anderson et al. (63)	Mild, moderate, severe	2–7 yrs	Severe TBI compared to mild and moderate TBI and controls showed poorer performances on sustained attention task (CPT)
Ornstein et al. (40)	Mild, moderate, severe	6–16 yrs	SADHD group showed slowed psychomotor speed (divided attention) at both 6-months and 12-months post-injury and more difficulty with dual task attention processes at 12-months post-injury.
Yeates et al. (28)	Moderate, severe	6–12 yrs	Severe TBI vs. OI but not moderate TBI vs. OI showed poorer performance on attention task (Underlining)
**OTHER NEUROPSYCHOLOGICAL TASKS**			
Studer et al. (35)	Mild	6–16 yrs	Mild TBI, but not OI, demonstrated reduced verbal learning associated with increased everyday attention problems.
Sinopoli et al. (37)	Mild, moderate, severe	7–17 yrs	SADHD exhibited a selective deficit in cancellation inhibition (response inhibition) compared to controls
Schachar et al. (38)	Mild, moderate, severe	5–17 yrs	SADHD with severe TBI showed longer stop signal reaction time (response inhibition)
Ornstein et al. (40)	Mild, moderate, severe	6–16 yrs	At 6 months post injury TBI-only and SADHD groups showed planning difficulty, PADHD children reached the solutions fastest and maintained the best performance overall; SADHD made fewer target hits (working memory) and slowed psychomotor speed (divided attention), the PADHD group demonstrated variable performance, while the TBI-only group maintained the best performance
Ornstein et al. (41)	Mild, moderate, severe	6–14 yrs	SADHD did not predict stop signal inhibition
Konrad et al. (53)	Moderate, severe	8–12 yrs	TBI without a SADHD showed slower stop signal task (response inhibition) compared to SADHD
Slomine et al. (36)	Severe	6–16 yrs	TBI-only showed reduced short-delay cued recall performance compared to SADHD.

*yr(s), year(s); SADHD, secondary ADHD; PADHD, primary ADHD; OI, orthopedic injury; CPT, continuous performance task*.

## Discussion

This scoping review summarized the state of the literature that assesses new-onset ADHD and attention problems that emerge after TBI in children and adolescents. Identified literature was organized into the following conceptual domains: prevalence, risk factors (divided into injury-related and non-injury related factors), associated behavioral features, and neuropsychological impairments.

The findings from the scoping review supports the increased prevalence of new clinically significant attention problems and SADHD following injury in severe TBI (28, 42, 48, 58), consistent with a recent meta-analysis of SADHD (24). The review also supports that there is a higher risk of new attention problems and SADHD in children and youth with low preinjury adaptive functioning (27, 31, 49, 63), and when the injury occurs in early childhood (<7 years of age at time of injury) (29, 42, 54, 59, 63). Evidence suggests that both sexes are equally vulnerable (27, 30, 36, 38, 40–42, 46, 48, 49, 52), and poor family functioning was sometimes (42, 45, 48) but not always (27, 32, 46, 49, 54) a risk factor for SADHD and new attention problems. These conclusions are supported by a recent review that specifically examined biopsychosocial factors associated with attention problems in youth with TBI (23). Due to the lack of topical limitation of a scoping review framework the current review also found evidence to support that commonly concurrent neuropsychological characteristics and behavioral presentation include: (1) co-occurring novel psychiatric problems including ODD, CD (27, 45, 49), and affective lability (27, 33, 47–50), (2) and reductions in daily functioning (communication, socialization, and adaptive functioning) (17, 40, 48). Furthermore, this scoping review identified mixed evidence as to: (1) the prevalence of new attention problems in mild TBI, (2) if children with SADHD are especially neuropsychologically impaired (37, 38, 40, 41, 53), (3) the predominant class of attentional impairment in youth with TBI due to inconsistent outcome measures (28, 60, 63), and (4) the role of brain lesions in attention problems after injury.

A wide range of prevalences were reported for SADHD and new attention problems after TBI which is likely influenced by the presence of identified risk factors in different study samples. Evidence from identified studies suggests that new attention problems and ADHD diagnoses may be more common when the injury occurs in early childhood (29, 42, 54, 59, 63). Children injured in early childhood may be most vulnerable to developing new attention problems after injury. This may be due to damage to brain circuitry that is responsible for basic attentional processes in early development or injury that prevents the future proper development of later maturing attentional processes (65, 66). Experiencing multiple injuries was associated with elevated risk for attention problems in the two reviewed studies that examined this and it should be an area for further study given that repeat injuries are common in children who play sports.

Though the prevalence of primary (i.e., developmental) ADHD is higher in males than females [3:1 (67)] this does not appear to be the case for ADHD secondary to TBI as the majority of studies reported no influence of sex. There is a need for additional research to determine the circumstances when mild TBI is a risk factor for secondary attention problems and ADHD as prevalence reported in these subjects was particularly varied. The majority of the reviewed studies included hospitalized children suggesting that they included more severe forms of mild TBI or children who suffered from other extensive injuries. This points to an underrepresentation of mild cases that are representative of typical mild cases of TBI that are seen in an emergency room, assessed by their general practitioner, or who receive no initial medical attention.

Studies that examined whether specific types of brain pathology detected with brain imaging are associated with new attention problems are difficult to synthesize due to their varied methodology. Specifically, the ability to meaningfully consider risk factors identified through neuroimaging is confounded by differing imaging modalities, time since injury, the severity of the injury, and population type in the identified studies. Some evidence supports the usefulness of imaging performed acutely after injury to inform the child's outcome including attention problems (27, 49, 56). However, one study reported acute imaging as a non-significant risk factor (48) and all positive studies were in hospitalized youth which brings into question the generalizability to those with mild injury. Currently, CT is most often performed to assess hematomas, brain swelling, and skull fracture though it is not recommended for mild injury to avoid unnecessary radiation exposure in children. MRI, which does not involve radiation, is more sensitive to certain types of pathology such as microhemorrhage, contusion, and gliosis, as well as axonal injury. MRI may be a more sensitive tool for prognosis through the detection of brain changes after TBI. However, there is currently no agreed-upon schema for coding of MRI findings used in clinical practice. It may be the case that diffuse axonal injury (68, 69), known to result from TBI, disrupts the functional integrity of widely distributed neural pathways that are involved in attention processes (70) and is associated with new attention problems, but this has not been assessed. Advanced MRI methods such as diffusion-weighted imaging that is sensitive to microscopic injury in the brain would be suitable diffuse axonal injury in attention circuits in future studies. Similarly, we did not identify any existing studies that use functional imaging (EEG, MEG, or fMRI) to examine circuit functional alterations that may predict new attention problems after injury.

Some studies include a comparison group with primary ADHD which could be informative for distinguishing related factors and may have implications for how the ADHD is managed. Whether SADHD mimics the primary ADHD endophenotype or not may inform whether these are separate disorders with different underlying mechanisms. The studies reviewed here demonstrated no associations between risk for secondary attention problems and family psychiatric history, unlike primary ADHD (71). This is consistent with a lack of a genetic association seen between genetic risk variants associated with primary ADHD and ADHD associated with TBI (72). The reviewed studies suggest that SADHD is associated with more debilitating outcomes (i.e., comorbid disorders, behavioral and functional impairments, and cognitive impairment) in some cases compared to primary ADHD. Of note treatment studies reviewed elsewhere suggest that treatment approaches that are beneficial for primary ADHD in children, methylphenidate, in particular, are also beneficial for treating SADHD (21, 22). Together these studies suggest that although attention problems that emerge after TBI may have separate underlying mechanisms than those that result from primary ADHD, existing treatment may be able to relieve symptoms through common overriding mechanisms.

This review has several important limitations. In general, there was a large amount of variability in the measures to assess similar study variables and outcomes which supports calls for the collection of TBI common data elements across all methodologies to more easily compare studies and draw conclusions (73). In this review, attention was assessed *via* diagnosis of ADHD as well as measures of attention (questionnaire and objective). The criteria for diagnosis are discrete, while measurements of self or parent-reported attention problems are heterogeneous in terms of their interpretation and reliability. This imposes limitations on the ability to characterize attention problems following TBI since the threshold on labeling behaviors as pathological is likely inconsistent. Another important source of methodological heterogeneity was that only half of the studies examined included a non-TBI comparison group, and half of these were an orthopedic injury group. The selection of an appropriate non-TBI comparison group controls for factors that predispose children to injury and are associated with the injury and recovery process. These factors are important to consider as they may influence the detection of the emergence of attention problems after TBI in children. Importantly, this review sought to explicitly examine new attention problems and SADHD however retrospective recall of preinjury functioning is potentially confounded. Recall can be heavily influenced by retrospective biases (26). Additionally, as per scoping review methodology, the quality of the included studies was not assessed. Therefore, there is a potential risk of bias inherent from included studies with potentially low-quality methodological design which limits the interpretations that can be made from this review. Finally, due to the large age range included in the majority of identified studies we were not able to break down the results by restricted age cohorts in order to identify age as a moderating factor between studies.

In summary, this scoping review synthesized a sizable body of evidence about the effects of TBI in childhood and adolescence on the emergence of new ADHD and attention problems. The results suggest that children that experience TBI are at increased risk for SADHD and new attention problems and that several injury and non-injury factors such as severity, the number of injuries, and age at injury may predict this risk. However, the evidence in some areas is lacking. This review identified that younger age of injury was a risk factor for new attention problems or SADHD within studies that included the youngest children (<7 years), however most studies included children across a large age range. Future studies should examine new attention problems in more restricted age cohorts that are expected to differ in their recovery from TBI due to many important neurodevelopmental changes take place across childhood. At the severe end of the TBI spectrum where the association with an increased risk for attention problems and SADHD is noted in this review and elsewhere (24) future clinical intervention studies can consider exploring psychosocial factors identified in this review. For example, interventions focused on family functioning and parental style, which have been associated with more attention problems (42, 45, 48), may prevent or attenuate the development of attention problems following injury. As even subclinical attention problems can affect learning and socializing in children the field would benefit from large studies of representative mild and moderate cases of TBI that measure attention problems dimensionally. These future studies should control for dimensional measures of preinjury attention problems and include appropriate control groups to understand the true risk for new attention problems following mild TBI. Further, longitudinal studies of representative cases will allow for the investigation of prevalence at various timepoints following injury to establish how often and in whom this type of impairment persists. Across severities a deeper understanding of the etiology of new attention problems and SADHD following brain injury can be achieved from the integration of advanced brain imaging. Functional and structural neuroimaging studies can shed light on the specific circuits that are disrupted by injury and cause attention problems. In addition to determining if or when mild TBI promotes new attention problems in children, these studies will be able to inform the mechanism by which these attention difficulties emerge and potentially distinguish them from the large existing body of literature on primary ADHD (74, 75). Evidence for prevalence, risk factors and characteristics of attention problems that develop after pediatric TBI is of interest to scientists as further understanding of the unique etiology of secondary attention problems will lead to knowledge of the mechanisms of symptom generation. This information may eventually be relevant to clinicians who treat these patients as this knowledge can aid the detection of attention problems and inform its management.

## Data Availability Statement

The original contributions presented in the study are included in the article/Supplementary Material, further inquiries can be directed to the corresponding author/s.

## Author Contributions

SS, AW, and RS contributed to conception and design of the study. SS and AW performed data screening and extraction and wrote the first draft of the manuscript. All authors contributed to the interpretation of the data, manuscript revision, read, and approved the submitted version.

## Funding

SS was supported by a Restracomp Scholarship from the Hospital for Sick Children and the Ontario Graduate Scholarship. AW was supported by the Scottish Rite Charitable Foundation of Canada.

## Conflict of Interest

The authors declare that the research was conducted in the absence of any commercial or financial relationships that could be construed as a potential conflict of interest.

## Publisher's Note

All claims expressed in this article are solely those of the authors and do not necessarily represent those of their affiliated organizations, or those of the publisher, the editors and the reviewers. Any product that may be evaluated in this article, or claim that may be made by its manufacturer, is not guaranteed or endorsed by the publisher.
