# Supplementary material for: A Systematic Scoping Review of New Attention Problems Following Traumatic Brain Injury in Children
Source: Front Neurol. 2021 Nov 10;12:751736. doi: 10.3389/fneur.2021.751736 (PMC8631327; doi:10.3389/fneur.2021.751736)
Supplement: Supplementary file 1 [file Table_1.docx]

**Electronic search strategy for PubMed**

| **#** | **Searches** | **Results** |
| --- | --- | --- |
| 7 | 6 and 4 and 5 | 159 |
| 6 | 1 or 2 or 3 | 139024 |
| 5 | (“ADHD” or “attention deficit hyperactivity’ or “attention deficit disorder”).mp. [mp=title, abstract, original title, name of substance word, subject heading word, floating sub-heading word, keyword heading word, organism supplementary concept word, protocol supplementary concept word, rare disease supplementary concept word, unique identifier, synonyms] | 30263 |
| 4 | (“secondary” or “after” or “following”).mp. [mp=title, abstract, original title, name of substance word, subject heading word, floating sub-heading word, keyword heading word, organism supplementary concept word, protocol supplementary concept word, rare disease supplementary concept word, unique identifier, synonyms] | 6290732 |
| 3 | (“concussion” or “concussions” or “concussive”).mp. [mp=title, abstract, original title, name of substance word, subject heading word, floating sub-heading word, keyword heading word, organism supplementary concept word, protocol supplementary concept word, rare disease supplementary concept word, unique identifier, synonyms] | 12293 |
| 2 | (“TBI” or “TBIs” or “mTBI” or “mTBIs”).mp. [mp=title, abstract, original title, name of substance word, subject heading word, floating sub-heading word, keyword heading word, organism supplementary concept word, protocol supplementary concept word, rare disease supplementary concept word, unique identifier, synonyms] | 25208 |
| 1 | (“brain injury” or “brain injuries” or “brain damage” or “head-injury” or “head injuries” or “head impact” or “head impacts”).mp. [mp=title, abstract, original title, name of substance word, subject heading word, floating sub-heading word, keyword heading word, organism supplementary concept word, protocol supplementary concept word, rare disease supplementary concept word, unique identifier, synonyms] | 129577 |
